# Maternal and neonatal outcome after vaginal breech delivery at term of children weighing more or less than 3.8 kg: A FRABAT prospective cohort study

**DOI:** 10.1371/journal.pone.0202760

**Published:** 2018-08-23

**Authors:** Lukas Jennewein, Ulrikke Kielland-Kaisen, Bettina Paul, Charlotte J. Möllmann, Anna-Sophia Klemt, Sally Schulze, Nina Bock, Wiebke Schaarschmidt, Dörthe Brüggmann, Frank Louwen

**Affiliations:** FRABAT FRAnkfurt Breech At Term Study Group; Department of Gynecology and Obstetrics, School of Medicine, Goethe-University, Theodor-Stern-Kai 7, Frankfurt, Germany; Centre Hospitalier Universitaire Vaudois, FRANCE

## Abstract

**Introduction:**

The clinical management of breech presentations at term is still a controversially discussed issue among clinicians. Clear predictive criteria for planned vaginal breech deliveries are desperately needed to prevent adverse fetal and maternal outcomes and to reduce elective cesarean section rates. The green-top guideline considers an estimated birth weight of 3.8 kg or more an indication to plan a cesarean section despite the lack of respective evidence.

**Objective:**

To compare maternal and neonatal outcome of vaginal intended breech deliveries of births with children with a birth weight of 2.5 kg– 3.79 kg and children with a birth weight of 3.8 kg and more.

**Design:**

Prospective cohort study.

**Sample:**

All vaginal intended deliveries out of a breech position of newborns weighing between 2.5 kg and 4.5 kg at the Obstetrics department at Goethe University Hospital Frankfurt from January 2004 until December 2016

**Methods:**

Neonatal and maternal outcome of a light weight group (LWG) (< 3.8 kg) was compared to and a high weight group (HWG) (≥ 3.8 kg) using Pearson’s Chi Square test and Fishers exact test. A logistic regression analysis was performed to detect an association between cesarean section rates, fetal outcome and the birth weight.

**Results:**

No difference in neonatal morbidity was detected between the HWG (1.8%, n = 166) and the LWG (2.6%, n = 888). Cesarean section rate was significantly higher in the HWG with 45.2% in comparison to 28.8% in the LWG with an odds ratio of 1.57 (95% CI 1.29–1.91, p<0.0001). In vaginal deliveries, a high birth weight was not associated with an increased risk of maternal birth injuries (LWG in vaginal deliveries: 74.3%, HWG in vaginal deliveries: 73.6%; p = 0.887; OR = 1.9 (95% CI 0.9–1.1))

**Conclusion:**

A fetal weight above 3.79 kg does not predict increased maternal or infant morbidity after delivery from breech presentation at term. Neither the literature nor our analyses document evidence for threshold of estimated birth weight that is associated with maternal and/or infant morbidity. However, patients should be informed about an increased likelihood of cesarean sections during labor when attempting vaginal birth from breech position at term in order to reach an informed shared decision concerning the birth strategy. Further investigations in multi center settings are needed to advance international guidelines on vaginal breech deliveries in the context of estimated birth weight and its impact on perinatal outcome.

## Introduction

The management of breech presentations at term is a controversially discussed topic in obstetrics. Several large cohort studies reported a lower neonatal morbidity and mortality when infants were delivered by planned cesarean section [1–4]. In contrast, numerous trials conducted in high resource settings showed a similar outcome of vaginal breech birth vs. planned cesarean section when patients were selected by stringent criteria, e.g. an obstetric conjugate (CVO) of more than 12 cm or an estimated fetal weight of 2.5 kg or more (5–12). Also, skilled practicioners and delivery in an upright position were identified as favorable settings for the vaginal birth from breech position. Complication rates were reported to be similar to cephalic deliveries [5].

Planned cesarean sections are increasing worldwide. Associated short—and long-term complications like hemorrhage, uterine rupture, and abnormal placentation parallel these rising procedure numbers [6–9]. Therefore, elective cesarean sections without reasonable medical indication need to be limited. Obstetricians require evidence based guidelines to recommend the most suitable delivery mode for every individual patient. By aiming to increase vaginal delivery rates maternal and fetal complications can be decreased. Currently, several selection parameters are part of national breech management guidelines: [10–12] The green-top guideline considers an estimated fetal weight above 3.8 kg a risk factor for vaginal delivery. The authors suggest cesarean section as the advisable mode of delivery [11]. However, clear evidence supporting this clinical management is currently insufficient.

The presented prospective cohort study includes 1054 participants who intended vaginal breech delivery at term and analyzes maternal and fetal outcomes when singletons with a birth weight of 2.5 kg to 3.79 kg (n = 888) were delivered compared to children weighing 3.8 kg (n = 166) and more. Because of an exclusion from the vaginal birth approach in most clinical management regiments we hypothesize an increased maternal and neonatal morbidity for children born out of a breech position weighing 3.8 kg– 4.49 kg.

## Materials and methods

### Patient cohort and patient selection

**Fig 1:**
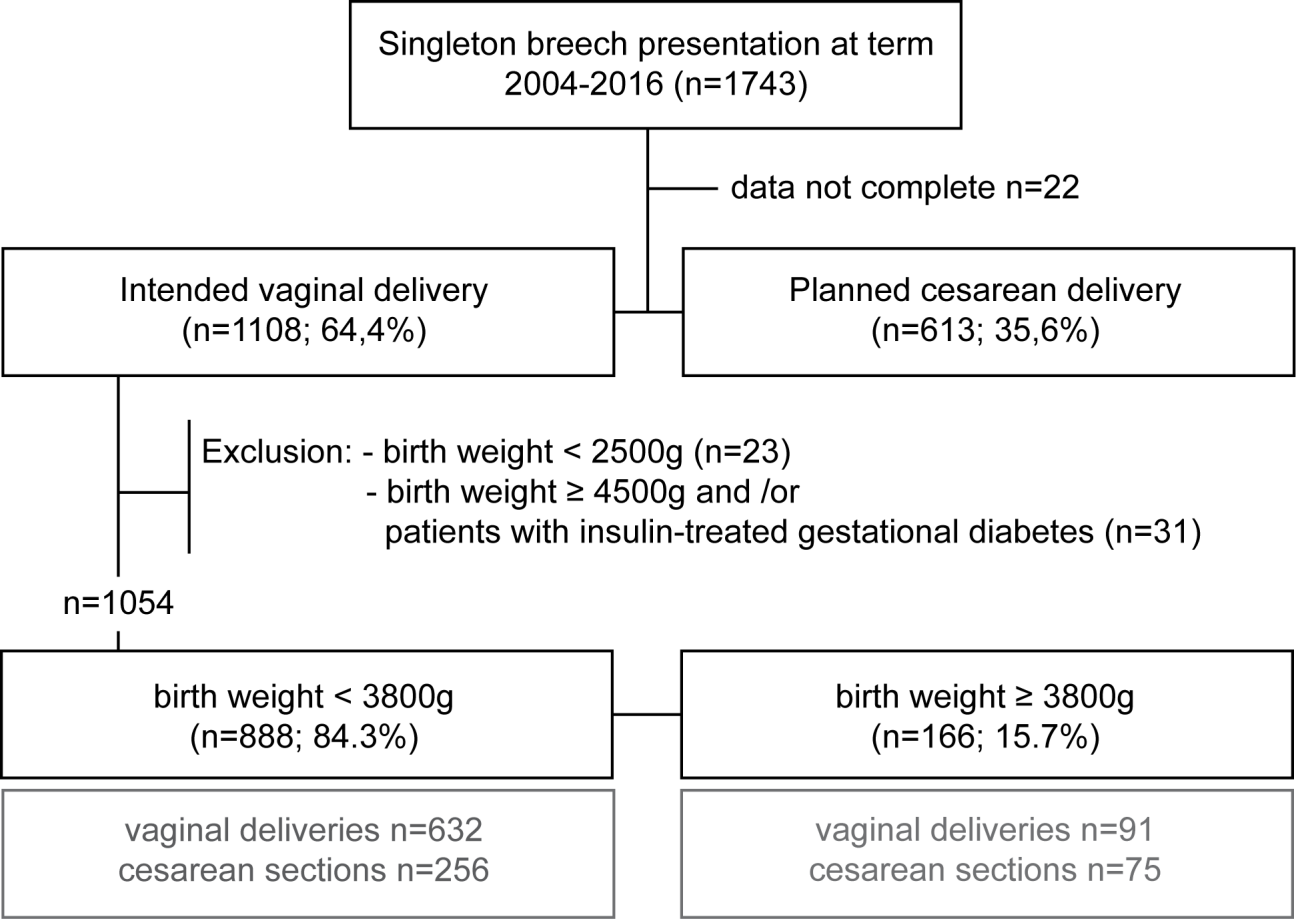
Flow chart representing study design and raw study numbers of cases included in this study.

A prospective cohort study was performed on the cohort of women presenting with breech at term (>37 weeks) at the Goethe University Hospital Frankfurt, Germany from January 2004 to December of 2016. The ethics committee of the Goethe University Hospital Frankfurt approved of the study protocol (Reference number 420/11). The requirement for patients informed consent was waived by the committee as all data was obtained within the hospitals standard care of patients intending a vaginal breech delivery. Patients received treatment as usual and only clinically relevant and required data was documented. Data was extracted from treatment files and anonymized by treating obstetricians who are part of the ethics committee approved study team. The ‘Perinatalerhebung Hessen’, a state database was used to obtain the data. Missing data, the mothers’ patient history as well as diagnoses and outcome parameters of neonates who were admitted to the neonatal intensive care unit (NICU) were gathered using the hospitals patient management system. All data was acquired and set into a table for analysis within this study after the patients were discharged. Of 1743 cases with a breech presentation at term 1054 expecting women intended a vaginal birth approach and did not meet exclusion criteria. Exclusion criteria were planned cesarean delivery (due to the reasons maternal wish, intrauterine growth restriction, malformations of the uterus, inability to perform spontaneous birth not related to the fetal position), insulin-treated diabetes, infant’s birth weight of over 4.49 kg or less than 2.5 kg. (Fig 1)

Women with breech presentation register for birth and delivery planning at the outpatient clinic at 34 to 36 weeks of gestation Vaginal delivery approach from breech position was offered in case of a successful previous vaginal birth or an obstetrical conjugate above 12 cm (measured by MRI) and an estimated birth weight of 2.5 kg or more of a proportional grown fetus. Weight of the fetus of ≥3.8kg, previous cesarean delivery or head flexion did not lead to exclusion.

Louwen et al. showed a superior maternal and fetal outcome from birth position on knees and arms or in upright position [5] The preferred birth position in all vaginal deliveries was on knees and arms or in upright position. In some cases, dorsal position was used to perform manual assisted delivery. In this study all birth positions were included.

### Statistical analyses

1054 women intended vaginal delivery from breech position and their babies weighed 2.5 kg to 4.49 kg at birth. There were no outliers. The Shapiro-Wilk-W-Test documents normal distribution of birth weights. (W = 0.987) 723 cases resulted in vaginal delivery. Within this group of successful vaginal births, the fetal weight was normal distributed (Shapiro-Wilk-W-Test W = 0.986) and there were no outliers.

Differences in birth weight-dependent variables e.g. duration of pregnancy is displayed only descriptively. Epidemiological data–for instance BMI and the mother’s age–was tested using student’s t-test with welch correction in cases of unequal variances.

Group differences in rates determining the fetal or maternal outcome as well as the calculated scores (modified PREMODA score) were tested in univariate contingency table based statistics using Pearson’s χ^2^ test and Fishers exact test.

The birth weight’s influence on outcome variables as well as the probability to receive a cesarean section was tested using a univariate logistic regression analysis.

Multivariate analyses were performed using a nonparametric Spearman’s ρ testing and are shown in a supplementary table (S1 File)

All statistical analyzes were carried out using JMP software (SAS Institute, Cary, USA).

## Results

We report of 1054 deliveries out of breech position. Mean fetal birth weight was 3351g. (Median = 3320g. 25% quartile = 3047.5g, 75% quartile = 3620g) To groups were generated according to fetal weight. 1) The low fetal weight group (LWG, 2.5 kg– 3.79 kg, n = 888) and 2) the high fetal weight group (HWG, 3.8 kg– 4.49 kg, n = 166). Maternal mean age (LWG: 32.2 years, HWG 32.2 years) and body mass index (BMI) of both groups were not significantly different, however the high birth weight group descriptively showed higher BMI values. (LWG: 23.0, HWG: 23.7, p = 0.05) Duration of pregnancy was significantly longer in the HWG (40.4 weeks respectively 39.4 weeks in the LWG). Parity was equally distributed in both groups as well as subtypes of breech positions. In both groups, the frank breech was the most common position. (Table 1)

**Table 1:** Vaginally intended deliveries out of a breech position at term, epidemiologics and maternal patient history characteristics, <3800g vs ≥3800g.

Characteristic	< 3800g (n = 888)	≥ 3800g (n = 166)	P value
**Age (mean, st.dev.)**	32.3 (4.5)	32.3 (4.3)	0.963
**BMI (mean, st.dev.)** ^**a**^	23.0 (2.8)	23.7 (4.4)	0.050
**Duration of pregnancy (mean, st. dev.)** ^**b**^	39.4 (1.2)	40.4 (0.9)	
**Parity** (n, %)			0.599
1	532 (59.9%)	97 (58.7%)	
2	216 (24.3%)	46 (27.5%)	
> 2	140 (15.8%)	23 (13.8%)	
**Gestational diabetes**	35 (3.9%)	5 (3.0%)	0.56
**Internal preconditions** ^**c**^	133 (15.0%)	18 (10.8%)	0.163
**Hemostasis disorder**	18 (2.0%)	5 (3.0%)	0.427
**Type of breech** (n, %)			0.634
Frank	540 (60.8%)	100 (60.2%)	
Complete	82 (9.2%)	10 (6.0%)	
Incomplete	92 (10.4%)	21 (12.7%)	
Footling	36 (4.1%)	6 (3.6%)	
Oblique Lie	5 (0.6%)	2 (1.2%)	
Missing data	133 (15.0%)	27 (16.3%)	
**Cesarean** (n, %, *OR, 95% confidence interval*)	255 (28.8%, *reference group*)	75 (45.2%, *2*.*0*, *1*.*5–2*.*9*)	< 0.0001
**Reason for cesarean** (*n, %*)	% of Cesarean	% of Cesarean	
Mothers whish	15 (5.9%)	5 (6.7%)	0.796
Delay in stage 1	82 (32.0%)	33 (44.0%)	0.056
Delay in stage 2	71 (27.7%)	24 (32.0%)	0.473
Abnormal fetal cardiography or doppler	102 (39.8%)	15 (20.0%)	0.002
Uterine scar or pathology	6 (2.3%)	0 (0.0%)	0.181
Placental reason	5 (2.0%)	1 (1.3%)	0.724
Cord prolapse	12 (4.7%)	2 (2.7%)	0.444
Bleeding or premature birth Indicators	3 (1.2%)	2 (1.5%)	0.342
Maternal reason	3 (1.2%)	0 (0.0%)	0.364
Percieved cephalopelvic disproportion	7 (2.7%)	3 (4.0%)	0.573
Chorioamnionitis	6 (2.3%)	3 (4.0%)	0.483
Other fetal reason	2 (0.8%)	0 (0.0%)	0.443

Vaginally intended deliveries out of a breech position at term, epidemiologics and maternal patient history characteristics, <3800g vs ≥3800g

^a^Missing BMI values: 30 patients

^b^Correlating variables, therefore only descriptive.

^c^Pre-existing hypertension, hypothyroidism, dermatologic diseases etc.

Cesarean section rate was significantly higher in the HWG with 45.2% in comparison to 28.8% in the LWG with an odds ratio of 2.0 (95% CI 1.5–2.9, p<0.0001) (see. Table 1 for subsample characteristics) Indications for cesarean section under labor were analyzed in the subgroup of cesarean sections after vaginal intended birth approach. A not significantly increased incidence of birth delays in stage one (LWG: 32.0%; HWG 44.0%, p = 0.056) was noted. Abnormal fetal cardiography or Doppler (LWG: 39.8%, HWG: 20%, p = 0.002) occurred significantly less in the HWG. (Table 1)

Comparing the outcome of newborns between the LWG and the HWG did not reveal a disadvantage for neonates weighing 3.8 kg or more. There was no difference between groups regarding the frequency of 5 minutes APGAR below 4 (LWG: 0.5%, HWG: 0.6%, p = 0,795), the stay on a neonatal intensive care unit (NICU) over 4 days (LWG: 4.6%, HWG: 4.8%, p = 0.886) or birth trauma (LWG: 0.8%, HWG: 1.2%). Moreover there were no differences in numbers of neurologic deficits (LWG: 0.7%, HWG: 1.2%, p = 0.471) or perinatal asphyxia (LWG: 1.5%, HWG 1.5%, p = 0.74). (Table 2).

**Table 2:** Vaginally intended deliveries out of a breech position at term, fetal outcome, <3800g vs ≥3800g.

Characteristic	< 3800g (n = 888)	≥ 3800g (n = 166)	P value	Odds Ratio (5%-95% confidence)
**APGAR 5‘ (n, %)**			0.131	
< 4	4 (0.5%)	1 (0.6%)	0.795	
4 < 7	21 (2.4%)	0 (0.0%)		
**NICU**			0.886	
> 4 days	41 (4.6%)	8 (4.8%)		
Up to 4 days	26 (2.9%)	6 (3.6%)		
**Intubation > 24h**	7 (0.8%)	1 (0.6%)	0.616	
**pH arterial blood < 7.0**	3 (0.3%)	3 (1.8%)	0.054*	
**Short time problems with breathing, bradycardia**	43 (4.8%)	8 (4.8%)	0.989	
**Birth trauma**	7 (0.8%)	2 (0.9%)	0.592	
**Neurologic deficits**	6 (0.7%)	2 (1.2)%	0.471	
**Perinatal asphyxia**	13 (1.5%)	3 (1.5%)	0.740	
**Deaths**	1^a^ (0.1%)	0 (0.0%)	0.665	
**Amniochorionitis**	29 (3.3%)	11 (6.6%)	0.046*	
**Umbilical cord complication**	91 (10.3%)	15 (10.1%)	0.227	
**Death or severe perinatal morbidity (PREMODA)**	50 (5.6%)	9 (5.6%)	0.914	0.96 (0.5–2.0)
**Death or severe perinatal morbidity potentially related to delivery mode**^**b**^	22^c^ (2.5%)	3^d^ (1.8%)	0.602	0.72 (0.2–2.4)

Vaginally intended deliveries out of a breech position at term, fetal outcome, <3800g vs ≥3800g.

*Fishers exact test

^a^1 Case of Anencephaly

^b^Excluded because morbidity criteria applied but the causes were not potentially related to delivery mode: 20 cases of amniochorionitis, 1 case of intracranial spontaneous bleeding after delivery without birth trauma or birth complication, 1 case of meconium ileus, 1 case of extensive hyperbilirubinemia, 1 case of intracranial infarction after birth without birth trauma, 1 case of spontaneous pneumothorax after birth, 1 case of preexisting intracranial cysts causing neurologic deficits, 1 case of arrhythmia associated with TRAK antibodies leading to an admission to the NICU of > 4 days, 1 case of cardiac malformation (ASD II°) leading to an admission to the NICU of > 4 days, 1 case of pneumonal artery stenosis, 1 case of G6DPH deficiency, 1 case of blood feto-maternal transfusion complication, 1 case of sleeping myoclonism

^c^9 cases of perinatal asphyxia, 8 cases of severe problems adapting after birth, 1 case of humerus fracture after assisted delivery of arms, 3 cases of plexus paralysis after assisted delivery of arms, 1 case of clavicular fracture after assisted delivery of arms

^d^1 case of severe problem adapting after birth, 1 case of perinatal asphyxia, 1 case of plexus paralysis after assisted vaginal birth

For a clear analysis a score adapted from the PREMODA study [12] was applied to analyze fetal morbidity. Neonates were labeled as cases with perinatal morbidity or mortality if at least one of the following criteria were met: death within 28 days after delivery, intubation > 24 h, 5’ APGAR < 4, stay on a NICU > 4 days, seizure < 24 h after birth or birth trauma excluding hematoma. Using this score, no differences between the HWG (1.8%) and the LWG (2.5%) have been detected. Cases of abnormalities that are unrelated to the delivery mode were excluded from these analyses (dysplasia, cases of amniochorionitis, neonatal infections). It results that neonates with a birth weight of 3.8 kg or more do not have a higher perinatal morbidity or mortality related to the delivery mode than lower weighing newborns with an OR of 0.72 (95% CI 0.2–2.4) in vaginally planned deliveries (Table 2).

A multivariate analysis of this cohort including fetal birth weight, delivery mode, pregnancy duration, parity and maternal BMI could not detect a correlation of those factors with fetal morbidity. The strongest correlation was found between fetal birth weight and pregnancy duration (ρ = 0.461 p>0.0001). However this analysis shows a positive correlation of the maternal BMI and the cesarean section rate (ρ = 0.115 p = 0.0002) (Figure A in S1 File).

We further analyzed all vaginal deliveries. (732 cases. Fetal weight mean was 3313g. Median = 3390g. 25% quartile = 3020g, 75% quartile = 3570g.) All cases of neonates with a birth weight of 2.5 kg– 3.79 kg (low weight group of vaginal deliveries, vLWG, n = 632) were compared to all cases of newborn weighting 3.8 kg– 4.49 kg (high weight group of vaginal deliveries, vHWG, n = 91).

Birth position in both groups were equally distributed. In 16.4%, patients delivered in dorsal position in the vLWG compared to 22% in the vHWG. In 43.2% a maneuver to assist the body and/or the head was necessary in the vLWG compared to 45.0% in the vHWG (no significant difference). The odds ratio for a manually assisted vaginal delivery was of 1.08 (95% CI 0.7–1.7) for neonates weighing 3.8 kg or more. (Table 3).

**Table 3:** Vaginal deliveries out of a breech position at term, fetal outcome, <3800g vs ≥3800g.

Characteristic	< 3800g (n = 632)	≥ 3800g (n = 91)	P value	Odds Ratio
**Birth position**			0.164	
Dorsal position	102 (16.4%)	20 (22.0%)		
Hands and knees / upright	530 (83.9%)	71 (78.0%)		
**Maneuvers necessary**	273 (43.2%)	41 (45.0%)	0.738	1.08 (0.7–1.7)
Help with body delivery	157 (24.8%)	23 (25.3)	0.929	
Classic maneuver	81 (12.8%)	15 (16.5%)	0.335	
Bickenbach	3 (0.5%)	0 (0.0%)	0.510	
Loveset or 180° torque	24 (3.8%)	3 (3.3%)	0.814	
Bracht	52 (8.2%)	4 (4.4%)	0.200	
Help with head delivery	233 (36.9%)	34 (37.4%)	0.927	
Suprapubic pressure	7 (1.1%)	1 (1.1%)	0.999	
Veith Smellie	147 (23.3%)	22 (24.2%)	0.847	
Frank nudge	89 (14.1%)	11 (12.2%)	0.633	
Other	24 (3.8%)	6 (6.7%)	0.205	
**APGAR 5‘ (n, %)**			0.254	
< 4	3 (0.5%)	1 (1.1%)		
4 < 7	15 (2.4%)	0 (0.0%)		
**NICU**			0.288	
> 4 days	27 (4.3%)	4 (4.4%)		
Up to 4 days	16 (2.5%)	5 (5.5%)		
**Intubation < 24h**	4 (0.6%)	1 (1.1%)	0.252	
**pH arterial blood < 7.0**	2 (0.3%)	3 (3.3%)	0.016^a^	
**Problems with adaptation**	28 (4.4%)	6 (6.6%)	0.362	
**Birth trauma**	7 (1.1%)	2 (2.2%)	0.381	
**Neurologic deficits**	7 (1.1%)	3 (3.3%)	0.20	
**Perinatal asphyxia**	11 (2.1%)	2 (2.2%)	0.759	
**Deaths**	1 (0.2%)	0 (0.0%)	0.704	
**Amniochorionitis**	18 (2.9%)	5 (5.5%)	0.179	
**Umbilical cord complication**	55 (8.7%)	11 (9.1%)	0.295	
**Death and severe morbidity (PREMODA)**	33 (5.2%)	5 (5.5%)	0.913	1.1 (0.4–2.8)
**Death and severe morbidity (PREMODA) potentially related to delivery mode**	16 (2.5%)^b^	3 (3.3%)^c^	0.670	1.3 (0.4–4.6)

Vaginal deliveries out of a breech position at term, fetal outcome, <3800g vs ≥3800g.

^a^In an analysis of the vHWG and a randomly picked sample of 91 datasets of the vLWG this test resulted in a p value above significance level. (data not shown)

^b^8 cases of perinatal asphyxia, 1 case of severe problem adapting after birth with a pulmonary hypertension, 1 case of isolated humerus fracture after assisted delivery of arms without asphyxia, 1 case of plexus paralysis and severe problems adapting after birth, 1 case of clavicular fracture of a child with a Prader-Willi-Syndrome, 1 case of respiratory adapting problems after birth, 2 case of a plexus paralysis after assisted delivery of arms without adapting problems where one of those children had remaining motoric handicap upon discharge, 1 case of cephalic fracture and perinatal asphyxia where a forceps had to be performed on the following head. No neurologic deficiencies at discharge.

^c^1 case of severe problem adapting after birth, 1 case of perinatal asphyxia, 1 case of plexus paralysis after assisted vaginal birth

Neonatal outcome analyses were performed using the same independent variables as mentioned above. Rates of birth trauma, long stays on the NICU (> 4 days), short time problems with adaption, intubation or perinatal asphyxia were not significantly different between vaginal birth groups. Calculating the severe morbidity and mortality rate using the modified PROMODA score (potentially associated with delivery mode) for both groups revealed no disadvantage for the vHWG (3.3%) in comparison to the vLWG (2.5%) (Odds ratio 1.3, 95% CI 0.4–4.6) (Table 3).

A birth weight of 3.8 kg or more was not associated with a higher rate of perineal tears (vLWG: 51.1%, vHWG: 52.8%; p = 0.473; OR = 1.2 (95% CI 0.8–1.8)); When grades of perineal tears were compared between weight groups though, the perineal tear grade III and IV rate was descriptively but not statistically significantly higher in the vHWG (vLWG: 1.6%, vHWG: 4.4%, p = 0.069, OR = 2.9 (95% CI 0.9–9.3)). There were no significant differences between groups regarding rates of vaginal or labial injuries, episiotomies or application of peridural anesthesia during labor. (Table 4)

**Table 4:** Vaginal deliveries out of a breech position at term, maternal outcome, <3800g vs ≥3800g.

Characteristic	< 3800g (n = 631)	≥ 3800g (n = 91)	P value	Odds ratio (5–95% confidence)
**Perineal injury**	309 (51.1%)	48 (52.8%)	0.473	1.2 (0.8–1.8)
1st° perineal tear	200 (31.7%)	23 (25.3%)	0.219	0.7 (0.4–1.2)
2nd° perineal tear	101 (16%)	22 (24.2%)	0.052	1.7 (1.0–2.8)
3rd° and 4th° Perineal tear	10 (1.6%)	4 (4.4%)	0.069	2.9 (0.9–9.3)
Episiotomies	17 (2.7%)	3 (3.3%)	0.743	1.2 (0.4–4.3)
Injury of the vagina or labia	188 (29.8%)	21 (23.1%)	0.189	0.7 (0.4–1.2)
PDA during birth	315 (55.4%)^a^	46 (57.5%)^b^	0.718	1.1 (0.7–1.8)

Vaginal deliveries out of a breech position at term, maternal outcome, <3800g vs ≥3800g.

^a^data of 62 cases missing

^b^data of 11 cases missing

A multivariate analysis including the following covariates within the group of successful vaginal deliveries was performed: Fetal birth weight, perineal injuries, fetal morbidity, maternal BMI, pregnancy duration, parity and the need to perform assisting maneuvers. Here we found a weak positive correlation of fetal morbidity and the need to perform assisting maneuvers (ρ = 0.153, p > 0.0001) and a weak correlation of perineal tears and fetal birth weight (ρ = 0.104, p = 0.005) (Figure B in S1 File)

**Fig 2:**
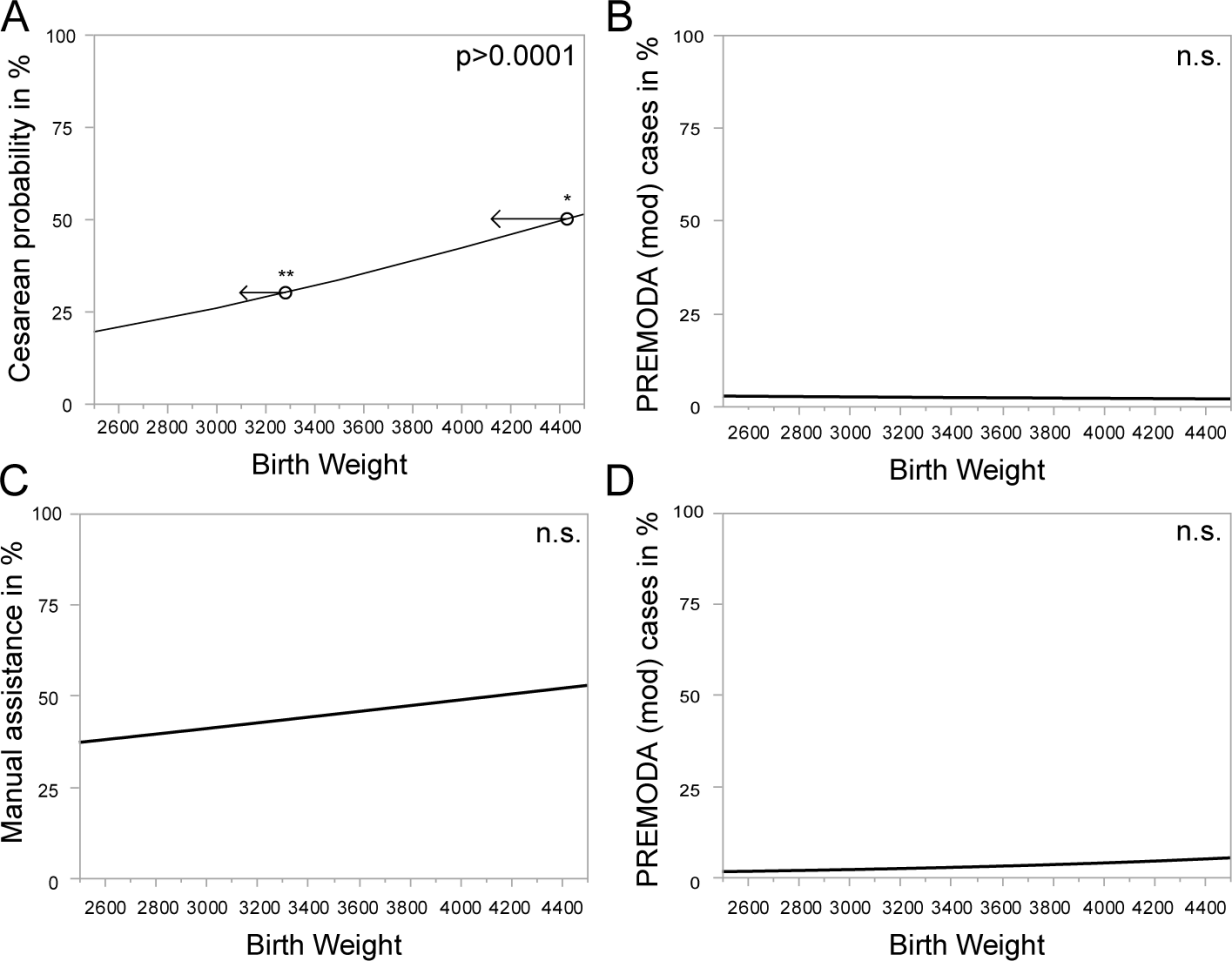
A) All vaginal intended deliveries included. Logistic regression of birth weight to cesarean sections. Y axis inverted to display probability indicated by the line. B) All vaginal intended deliveries included. Logistic regression of birth weight to percentage of PREMODA cases (potentially associated with delivery mode). Y axis inverted to display probability indicated by the line. C) All vaginal deliveries included. Logistic regression of birth weight to manually assisted vaginal births in %. Y axis inverted to display probability indicated by the line. D) All vaginal deliveries included. Logistic regression of birth weight to percentage of PREMODA cases (potentially associated with delivery mode). Y axis inverted to display probability indicated by the line.

To test if not only the through guidelines proposed and therefore selected cut off of 3.8 kg showed differences in the clinical outcome, a logistic regression analyses of all vaginally planned breech deliveries and vaginal births were performed. The likelihood of cesarean sections increased with higher birth weight (Odds ratio: 1.074 per 100 gram birth weight addition. (p>0.0001)). (Fig 2A) Accordingly a birth weight above 4433g implies a likelihood of cesarean section of 50% and more. A 30% cesarean rate is met with a birth weight of 3283g. No significant association between the birth weight and neonatal morbidity and mortality using the modified PREMODA score (potentially associated with delivery mode) was detected (Odds ratio: 1.017 per 100 gram birth weight addition. p = 0.736). (Fig 2B) Analyzing all vaginal deliveries, the likelihood of manual assistance was not significantly associated with increasing birth weight (Odds ratio: 1.032 per 100 gram birth weight addition (p = 0.0949)). (Fig 2C) The modified PREMODA score (potentially associated with delivery mode) did not show a correlation with birth weight (Odds ratio: 1.064 per 100g birth weight addition, p = 0.260) (Fig 2D).

All statistical analyzes were additionally performed with equal sample sizes. We compared the HWG / vHWG to a subgroup of automatized randomly chosen 166 (HWG) / 91 (vHWG) cases out of the respective control group. Here, no other results have been obtained with regards to content if not indicated otherwise within the respective Fig (Data not shown)

## Discussion

Obstetric management of breech presentation at term is not uniform and up to now it is a controversially discussed issue whether a cesarean or a vaginal delivery is to be recommended. The reasons are missing evidence based guidelines and contradicting publications about this matter. E.g. the Term Breech Trial by Hannah *et al* [13] found vaginal breech deliveries to have a higher morbidity rate compared to planned cesarean sections. In the aftermath study methods were criticized and opposing results were published [14–18]. Meanwhile the opinion that vaginal delivery can be recommended if certain criteria are met is emerging. A consistent international recommendation is still missing. Several different characteristics are used as exclusion criteria for vaginal planned breech deliveries. The English breech guideline–green-top guideline–uses a birth weight of 3.8 kg or more as an indication for a cesarean section [11]. In order to validate this limit we report of 1054 vaginal planned breech deliveries at term evaluated in a prospective cohort study.

Evaluating all deliveries and their fetal outcomes using a modified PREMODA score, a higher morbidity for children weighing over 3.79 kg could not be shown. Neither analyzes of all vaginal indented deliveries out of a breech position at term (Table 2) nor analyzes of all successful vaginal breech deliveries at term (Table 3) showed a higher morbidity or mortality rate for the respective infants with higher birth weights. When logistic regression analysis was performed to cave out any correlation of perinatal fetal morbidity with birth fetal birth weight no association was found. Even the rates of mother’s birth injuries in vaginal deliveries from breech position at term were not significantly higher in the vHWG compared to the vLWG. This falsifies our initially presumed hypothesis of a positive correlation of maternal and neonatal morbidity with fetal birth weight. We here conclude that an upper weight limitation for vaginal breech birth approaches is not necessary when fetal or maternal morbidity is taken into account.

To quantify adverse outcomes of neonates, a modified PREMODA score was used. All single cases covered by this score were looked upon in detail. This excluded cases with adverse outcomes where the mode of delivery was not relevant to the neonate’s condition. Some hereditary conditions are associated with macrosomia or growth restriction, posing as a possible bias–these cases were excluded in order to purify our analysis of perinatal morbidity.

We saw a non-significant tendency for heavier children to produce more higher-graded perineal tears. A higher birth weight is known to be associated with a higher rate of perineal tears in cephalic deliveries. [19] Here, in international guidelines no strict recommendation to plan a cesarean section is given in cases of expected macrosomia. [20] In vaginal breech presentation this should also not lead to the recommendation of a cesarean since advantage of the vaginal birth is still predominant.

The likelihood to result in cesarean section correlates positively with the birth weight in vaginal planned deliveries. (Table 2, Fig 2) Women with an estimated birth weight of their expected newborn above 4.4 kg have a probability to receive a cesarean section during labor of more than 50%. With an odds ratio of 1.074 per 100 gram this association is quite weak and other parameters also could be responsible for increasing cesarean section rates in deliveries with newborn of higher birth weights. The birth weight correlated positively with pregnancy duration. Here, also deliveries with medically induced labor were included in our analysis. Since induced birth can be associated with a higher probability of cesarean sections [21,22], this might influence this statistical analysis. The influence of fetal birth weight on cesarean section rates is well known in cephalic deliveries [23] and thereby is not surprising in our breech cohort.

The role of the maternal BMI, pregnancy duration and parity in obstetrics and perinatal morbidity is well known [24–26]. When we analyzed those factors within a multivariate analysis in our cohort (S1 File), we found that maternal BMI and pregnancy duration correlated positively with cesarean section rates while parity correlated negatively with cesarean section rates–confirming the known literature. This analysis shows that not only the fetal birth weight but also other factors are responsible for elevated cesarean section rates in our cohort.

A recommendation to plan cesarean section in advance is not indicated in breech presentations at term with an estimated fetal birth weight of over 3.79 kg since perinatal morbidity does not differ. Nevertheless likelihood to receive a cesarean should be discussed with expecting women. The breech position of the baby is as itself a reason for an elevated cesarean section rate compared to cephalic deliveries which is due to limited options to accelerate birth in cases of birth delay or abnormal fetal cardiography. In cephalic deliveries, e.g. a micro-blood analysis can be performed to exclude fetal acidosis or an operational vaginal delivery can be performed to accelerate birth. As in breech deliveries comparable methods are not established leading to increased cesarean section rates, expecting women have to be informed. The cesarean section rate probabilities and their changes due to a) the fetal position and b) the birth weight should be addressed in birth mode planning medical appointments.

It has been shown that cesarean section after onset of labor is favorable compared to planned cesarean section in regard of long term fetal outcome. [27] Therefore we provide secondary cesarean section after spontaneous initiation of contractions in our center in Frankfurt for women who undergo planned cesarean section due to medical reasons or mother’s wish. Women should be aware of their options, probabilities and the respective outcome before making a decision regarding their delivery mode. When pregnant women present their selves at term with a breech position and an estimated birth weight of over 3.79 kg, a vaginal birth approach can be offered (if not contraindicated otherwise). The elevated probability to receive a cesarean section and whether this has a consequence on the decision about the intended delivery mode should be discussed with expecting women.

A weakness of our study method might be that the estimated birth weight and the actual birthweight can differ up to 15% [28] and is examiner-dependent. Pregnant women have to be advised regarding their mode of delivery based on those unprecise values but this study relies on actual birth weight measurements. Of note, it has been reported that the intrauterine position of the fetus (cephalic or breech presentation) does not seem to influence the accuracy of sonographic estimated birth weight measurements [29]. Importantly, the conclusion from this study that a fetal birth weight above 3.8 kg does not enhance likelihood of adverse fetal outcome will release tension and insecurity about the intended delivery mode in cases when the estimated birth weight lies 15% underneath 3.8 kg.

Another limitation of this study is that the acquired data is based on a single center. Since quality standards in obstetric departments and regional cesarean section rates are differing quite largely, some of our findings (mostly concerning cesarean section rates) may not be applicable or reproducible in any other hospital or country. The outcome of vaginal deliveries out of a breech position depends on the obstetrician’s expertise. In order to implement vaginally intended breech deliveries into clinical practice it needs more education and a growing number of hospitals offering a vaginal birth approach.

This study gives detailed analyses of a large collective of breech deliveries at term in our center in Frankfurt from 2004 until 2016. However the data is based on one center only impact on guideline development and clinical practice may be limited. Randomized controlled trials with large patient cohorts in multi-center settings are needed in order to supply data for evidence based guidelines.

## Supporting information

S1 File Multivariate analysis. Weak correlation indicated by brown coefficient (from 0.1 to 0.29) Moderate correlation indicated by red coefficient (from 0.3 to 0.49) 30 BMI values are missing. Figure A) Multivariate analysis of all vaginally intended deliveries (n = 1053) including the following variables: Pregnancy duration (days), maternal BMI (kg/m^2^), parity, mod. PREMODA Score, fetal birth weight (g). Figure B) Multivariate analysis of all successful vaginal deliveries (n = 723) including the following variables: perineal injury (yes or no), fetal birth weight (g), mod. PREMODA Score, assisted delivery (manual assistance necessary: yes or no), maternal BMI (kg/m^2^), pregnancy duration (days), parity. Spearman’s ρ coefficient and p values are indicated.
(DOCX)
