# Supplementary material for: Maternal and neonatal outcome after vaginal breech delivery at term of children weighing more or less than 3.8 kg: A FRABAT prospective cohort study
Source: PLoS One. 2018 Aug 23;13(8):e0202760. doi: 10.1371/journal.pone.0202760 (PMC6107207; doi:10.1371/journal.pone.0202760)
Supplement: S1 File — Weak correlation indicated by brown coefficient (from 0.1 to 0.29) Moderate correlation indicated by red coefficient (from 0.3 to 0.49) 30 BMI values are missing. Figure A) Multivariate analysis of all vaginally intended deliveries (n = 1053) including the following variables: Pregnancy duration (days), maternal BMI (kg/m2), parity, mod. PREMODA Score, fetal birth weight (g). Figure B) Multivariate analysis of all successful vaginal deliveries (n = 723) including the following variables: perineal injury (yes or no), fetal birth weight (g), mod. PREMODA Score, assisted delivery (manual assistance necessary: yes or no), maternal BMI (kg/m2), pregnancy duration (days), parity. Spearman’s ρ coefficient and p values are indicated. (DOCX) [file pone.0202760.s001.docx]

**S1 File Multivariate analysis**

**A) Vaginally intended deliveries (n=1054)**

| **Variable** | **mit Variable** | **Spearman ρ** | **p > \|ρ\|** |
| --- | --- | --- | --- |
| Pregnancy duration | Maternal BMI | 00847 | 0.0067 |
| Parity | Maternal BMI | -0.0332 | 0.2890 |
| Parity | Pregnancy duration | -0.1048 | 0.0007 |
| Mod. PREMODA score | Maternal BMI | 0.0312 | 0.3188 |
| Mod. PREMODA score | Pregnancy duration | 0.0192 | 0.5333 |
| Mod. PREMODA score | Parity | -0.0283 | 0.3580 |
| Fetal birth weight | Maternal BMI | 0.1500 | <.0001 |
| Fetal birth weight | Pregnancy duration | 0.4605 | <.0001 |
| Fetal birth weight | Parity | 0.0643 | 0.0368 |
| Fetal birth weight | Mod. PREMODA score | -0.0022 | 0.9431 |
| Vaginal delivery | Maternal BMI | -0.1149 | 0.0002 |
| Vaginal delivery | Pregnancy duration | -0.1044 | 0.0007 |
| Vaginal delivery | Parity | 0.2176 | <.0001 |
| Vaginal delivery | Mod. PREMODA score | 0.0249 | 0.4200 |
| Vaginal delivery | Fetal birth weight | -0.1353 | <.0001 |

**B) Successful vaginal deliveries (n=723)**

| **Variable** | **mit Variable** | **Spearman ρ** | **p > \|ρ\|** |
| --- | --- | --- | --- |
| Mod. PREMODA score | Perineal injury | -0.0066 | 0.8593 |
| Fetal birth weight | Perineal injury | 0.1041 | 0.0051 |
| Fetal birth weight | Mod. PREMODA score | 0.0443 | 0.2339 |
| Assisted delivery | Perineal injury | 0.0500 | 0.1795 |
| Assisted delivery | Mod. PREMODA score | 0.1526 | <.0001 |
| Assisted delivery | Fetal birth weight | 0.0717 | 0.0540 |
| Maternal BMI | Perineal injury | 0.0557 | 0.1412 |
| Maternal BMI | Mod. PREMODA score | 0.0580 | 0.1254 |
| Maternal BMI | Fetal birth weight | 0.1281 | 0.0007 |
| Maternal BMI | Assisted delivery | 0.0330 | 0.3839 |
| Pregnancy duration | Perineal injury | 0.0862 | 0.0205 |
| Pregnancy duration | Mod. PREMODA score | 0.0252 | 0.4998 |
| Pregnancy duration | Fetal birth weight | 0.4333 | <.0001 |
| Pregnancy duration | Assisted delivery | 0.0934 | 0.0121 |
| Pregnancy duration | Maternal BMI | 0.0666 | 0.0786 |
| Parity | Perineal injury | -0.0707 | 0.0573 |
| Parity | Mod. PREMODA score | -0.0390 | 0.2946 |
| Parity | Fetal birth weight | 0.1365 | 0.0002 |
| Parity | Assisted delivery | -0.1210 | 0.0011 |
| Parity | Maternal BMI | 0.0028 | 0.9412 |
| Parity | Pregnancy duration | -0.0694 | 0.0623 |

.
